# Latent class analysis and machine learning for clinical subtyping prediction and differentiation in suspected neurosyphilis patients

**DOI:** 10.3389/fcimb.2025.1665468

**Published:** 2025-11-25

**Authors:** Sirui Wu, Yike Huang, Lan Luo, Jielun Deng, Yuanfang Wang, Fei Ye, Dongdong Li

**Affiliations:** Department of Laboratory Medicine, West China Hospital of Sichuan University, Chengdu, China

**Keywords:** neurosyphilis, latent class analysis, subtyping, machine learning, cerebrospinal fluid biomarkers

## Abstract

**Objective:**

Neurosyphilis presents significant diagnostic and therapeutic challenges due to its heterogeneous clinical manifestations, absence of a gold-standard diagnostic criterion, and variable treatment responses. This study aims to identify clinically homogeneous subtypes of suspected neurosyphilis patients and develop a machine learning-based subtyping model to support clinical decision-making.

**Methods:**

Data from 451 suspected neurosyphilis patients were retrospectively collected from West China Hospital of Sichuan University. Patients were divided into a model development cohort (n=369) and an external validation cohort (n=82) by time. Latent class analysis (LCA) was performed to identify subtypes, with the optimal class number determined by model fit indicators. Key predictive variables were selected using LASSO regression and Boruta algorithm. Six machine learning algorithms were employed to build LCA subtype prediction models. Feature importance was interpreted via SHAP analysis, and model generalizability was assessed using the external cohort.

**Results:**

LCA classified patients into three homogeneous subtypes: “typical neurosyphilis” (43.7%; predominantly male, high serum TRUST titer, significant CSF abnormalities, and robust intrathecal immune activation), “atypical neurosyphilis” (17.9%; absence of elevated CSF protein, mild intrathecal IgG synthesis), “non-neurosyphilis” (38.5%; normal CSF parameters). Six variables (age, serum TRUST titer, CSF protein, CSF nucleated cells, IgG index, CSF TTs) were used for model construction. The XGBoost model demonstrated optimal performance, achieving an AUC of 0.966 (accuracy: 87.3%) on the internal test set and 0.970 (accuracy: 91.5%) on the external validation set. Key predictors included CSF nucleated cells, CSF TTs, and IgG index.

**Conclusion:**

This study defines three clinically meaningful latent subtypes of neurosyphilis. The developed XGBoost model effectively discriminates between these subtypes of neurosyphilis and non-neurosyphilis in clinical settings, facilitating timely diagnosis and treatment.

## 1 Introduction

Neurosyphilis, a severe complication arising from syphilis due to the invasion of the central nervous system (CNS) by *Treponema pallidum*, can lead to diverse neuropsychiatric manifestations and irreversible neurological damage (Ropper, 2019). The World Health Organization estimates approximately 8 million new adult syphilis cases globally in 2022 (Rowley et al., 2019), with a notable resurgence observed post-COVID-19 pandemic (Soriano et al., 2023). Although systematic surveillance data on neurosyphilis incidence remain limited, the rising diagnostic rates of syphilis suggest a parallel increase in neurosyphilis burden, posing substantial public health challenges (Singh, 2020).

*Treponema pallidum* can invade the CNS in the early stage of primary infection, progressing to asymptomatic or symptomatic neurosyphilis, with the latter categorized into syphilitic meningitis, meningovascular syphilis, general paresis, and tabes dorsalis based on the neuroanatomical involvement (Ropper, 2019). The diagnosis of neurosyphilis, however, remains impeded by nonspecific clinical presentations and the absence of gold-standard diagnostic criteria, while these conventional classifications exhibit limitations in informing therapeutic decisions (Chen et al., 2025). Current diagnostic reliance on serological tests, cerebrospinal fluid (CSF) analysis, and epidemiological data often fails to capture the disease’s complex pathophysiology and individualized progression patterns. While intravenous aqueous penicillin G (18–24 million units daily for 10–14 days) remains the recommended therapy, treatment responses show marked heterogeneity (Workowski et al., 2021). Early intervention may mitigate cognitive decline (Davis et al., 2021), yet evidence demonstrates inverse correlations between baseline CSF protein levels and subsequent cognitive improvement or CSF-VDRL titer reduction (Roberts and Emsley, 1995). Notably, patients with CSF pleocytosis or parenchymal forms (general paresis, tabes dorsalis) exhibit poorer cognitive recovery post-treatment compared to those without, underscoring the prognostic significance of subtypic variability (Davis et al., 2021; Chen et al., 2025).

The precision subtyping may help address these diagnostic and therapeutic challenges. While genotyping of *Treponema pallidum* has been established and correlates with neurosyphilis susceptibility, systematic characterization of clinically meaningful host-derived subtypes remains lacking (Marra et al., 2010). Latent class analysis (LCA) offers a robust solution for identifying subgroups with shared characteristics, which has been widely applied in mental health research (Kongsted and Nielsen, 2017) and is increasingly employed in infectious disease studies (Doolan et al., 2021; Veličko et al., 2022). Concurrently, machine learning (ML) has demonstrated remarkable utility in data-intensive clinical microbiology applications (Peiffer-Smadja et al., 2020). Previous studies have developed predictive models for neurosyphilis diagnosis. Zou et al. collected clinical characteristics and laboratory data to train an eXtreme Gradient Boosting (XGBoost) model for predicting the diagnostic outcomes of neurosyphilis, demonstrating its good and generalizable performance (Zou et al., 2023). Li et al. developed a novel Random Forest (RF)-based classifier utilizing proteomic data to identify potential biomarkers for classifying neurosyphilis patients (Li et al., 2024). Building upon these advances, this study seeks to further refine neurosyphilis classification through a combined LCA and ML approach.

This study aims to: (1) identify clinically distinct neurosyphilis subtypes through LCA, (2) characterize inter-subtype biomarker differences, and (3) develop interpretable ML models using these subtypes as outcome categories to enhance diagnostic precision and subtypic classification. This subtype-to-diagnostic pipeline holds the potential to enable precise management of neurosyphilis grounded in subtype-specific mechanistic insights.

## 2 Materials and methods

### 2.1 Participants and study design

This retrospective study enrolled 451 patients with suspected neurosyphilis at West China Hospital, Sichuan University, from October 2019 to September 2024. Suspected neurosyphilis was defined as either: (1) seropositivity for *Treponema pallidum* particle agglutination assay (TPPA) with concomitant CNS symptoms, or (2) serum TPPA positivity with serum toluidine red unheated serum test (TRUST) titer ≥1:16 without CNS symptoms. CNS manifestations included hemiplegia, aphasia, seizures, lower limb weakness, muscle atrophy, papilledema, neck stiffness, diplopia, ptosis, ataxia, amnesia, impaired judgment/memory, cognitive dysfunction, mood alterations, and personality changes. Patients living with HIV were excluded.

The study population was divided into two cohorts by time: Cohort 1 (n=369, October 2019-December 2023) for LCA model development and ML training, and Cohort 2 (n=82, January-September 2024) for LCA model development and ML external validation. The study protocol received ethical approval from the Institutional Review Board of West China Hospital.

### 2.2 Data acquisition and preprocessing

Demographic, clinical, and laboratory data were extracted from electronic medical records. Variables with ≥40% missing values were excluded, retaining 18 variables for initial analysis. Highly correlated variables (correlation coefficient >0.65) were eliminated through correlation analysis (**Supplementary Figure S1**). Continuous variables for LCA were dichotomized using diagnostic thresholds derived from clinical standards, receiver operating characteristic (ROC) analysis, or literature evidence (see Appendix). The variable selection for ML incorporated least absolute shrinkage and selection operator (LASSO) regression, Boruta algorithm, statistical significance testing, correlation analysis, and clinical relevance assessment (**Supplementary Figure S2**, **Supplementary Figure S3**, **Supplementary Table S2**).

### 2.3 Latent class analysis

LCA was performed using the R poLCA package (Drew A. Linzer, 2011) with seven key variables (See supplementary materials for threshold derivations): sex (male=1), serum TRUST titer (≥1:16 = 1), CSF treponemal tests (TTs) (reactive=1), CSF non-treponemal tests (NTTs) (reactive=1), CSF protein (≥0.5g/L=1), albumin quotient (≥0.007138 = 1), and IgG synthesis rate (≥5.81 = 1). Models with 2–5 latent classes were evaluated using Akaike information criterion (AIC), Bayesian information criterion (BIC), likelihood, entropy, Lo-Mendell-Rubin (LMR) test, and bootstrap likelihood ratio test (BLRT), with optimal class number determined by statistical fit and clinical interpretability.

### 2.4 Machine learning

Six ML algorithms - RF, XGBoost, Gradient Boosting Decision Tree (GBDT), Support Vector Machines (SVM), Logistic Regression (LR), and Artificial Neural Network (ANN) - were evaluated for subtype classification. Cohort 1 was randomly split into a training set (70%) and a test set (30%). Model development employed 10-fold cross-validation with grid search hyperparameter tuning. Performance metrics included area under the ROC curve (AUC), accuracy, kappa, sensitivity, specificity, positive predictive values (PPV), negative predictive values (NPV), calibration curves, and decision curve analysis. The optimal algorithm underwent SHapley Additive exPlanations (SHAP) analysis for feature importance interpretation using the R fastshap (Greenwell B, 2024) and shapviz (Mayer M, 2025) packages, with external validation performed on cohort 2.

### 2.5 Statistical analysis

Statistical analyses utilized R version 4.4.1 (R Core Team, 2024) and IBM SPSS Statistics for Windows, version 24.0 (IBMCorp., Armonk, N.Y., USA). Normally distributed continuous variables were reported as mean ± SD, non-normal variables as median (lower quartile, upper quartile), and categorical variables as frequencies (proportion%). The t-tests (normal distribution), Kruskal-Wallis tests (non-normal distribution), or chi-square tests (categorical variables) for group comparisons were employed; the Fisher’s exact test for correlation analysis was employed, with statistical significance set at *P* < 0.05.

## 3 Results

### 3.1 Latent class analysis of suspected neurosyphilis patients

Latent class analysis, incorporating seven key variables (sex, serum TRUST titer, CSF TTs, CSF NTTs, CSF protein, albumin quotient, and IgG synthesis rate), identified optimal subtypic clustering among suspected neurosyphilis patients. Comparative evaluation of models with two–five latent classes (**Table 1**) excluded the five-class solution due to one subgroup comprising <10% (8.9%) of the population. The three-class model demonstrated superior statistical properties, evidenced by lower AIC and BIC values, adequate entropy, clinically plausible class distribution, and strong theoretical interpretability.

**Table 1:** Model fit statistics for latent class models by class number.

Classes	AIC	BIC	Likelihood	Entropy	LMR test	BLRT
2	3632.655	3694.327	-1801.328	0.815	<0.001	0.10
3	3547.981	3642.544	-1750.990	0.849	<0.001	0.03
4	3477.519	3604.975	-1707.760	0.829	<0.001	0.62
5	3483.473	3643.820	-1702.736	0.841	0.009	0.24

AIC, Akaike information criterion; BIC, Bayesian information criterion; LMR, Lo-Mendell-Rubin; BLRT, bootstrapped likelihood ratio test.

**Figure 1:**
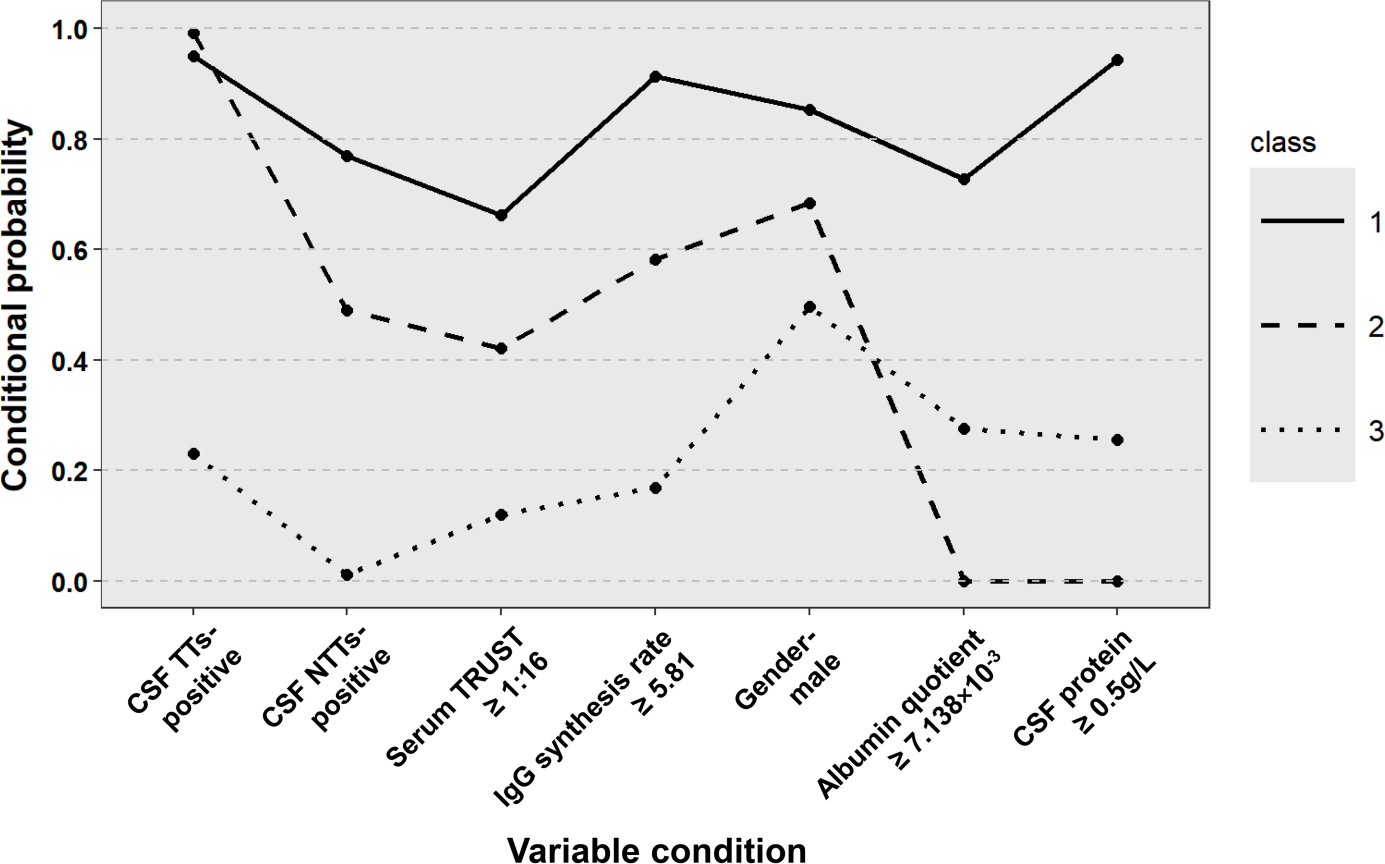
Distribution of potential categories of patients with suspected neurosyphilis. CSF, cerebrospinal fluid; TTs, treponemal tests; NTTs, non-treponemal tests; TRUST, toluidine red unheated serum test.

The final classification comprised three distinct classes: Class 1 (43.7%) represented typical neurosyphilis characterized by male predominance, high serum TRUST titers (≥ 1:16), marked CSF abnormalities, blood-brain barrier (BBB) disruption, and a significant increase in intrathecal IgG synthesis. Class 2 (17.9%) comprised atypical neurosyphilis cases showing normal CSF protein levels but demonstrable mild intrathecal IgG production. Class 3 (38.5%) included non-neurosyphilis patients with negative CSF treponemal antibodies and normal CSF indicators. Conditional probability distributions revealed significant differentiation across all seven input variables, with particularly strong discrimination observed in CSF protein (Class 1: 94.28% probability of positivity vs Class 3: 25.57%) and IgG synthesis rate (Class 2: 58.14% vs Class 3: 16.87%) (**Figure 1**).

### 3.2 Clinical characteristics classified by latent class

External validation of the latent class classification was performed by assessing differences in clinical indicators not included in the original LCA. As presented in **Table 2**, no statistically significant differences were observed in sex distribution or serum IgG levels across the three classes. However, indicators directly associated with neurosyphilis diagnosis (serum TRUST titers and CSF NTTs) and indicators reflecting intrathecal humoral immune activation and BBB impairment (CSF albumin, CSF IgG, IgG quotient, albumin quotient, IgG index, and IgG synthesis rate) exhibited significant interclass variations (*P*<0.001). Besides, CSF TTs, CSF chloride, and clinical diagnosis demonstrated no significant divergence between Class 1 (typical neurosyphilis) and Class 2 (atypical neurosyphilis); CSF protein concentrations and nucleated cell counts did not differ significantly between Class 2 and Class 3 (non-neurosyphilis). These findings validate the clinical relevance of the LCA-derived subtypes.

**Table 2:** Demographic, clinical, and laboratory features of subjects stratified by latent classes.

Variables	Class 1	Class 2	Class 3	*P*
N	192	76	183	
Age (y)	51.50 (42.00, 58.00)	47.50 (36.25, 54.00)	51.00 (36.00, 41.30)	0.024^a^
Gender				0.509
Male	131 (68.2%)	49 (64.5%)	131 (71.6%)	
Female	61 (31.8%)	27 (35.5%)	52 (28.4%)	
Serum TRUST titer				<0.001^a,b,c^
≥1:16	130 (67.7%)	38 (50.0%)	17 (9.3%)	
<1:16	62 (32.3%)	38 (50.0%)	166 (90.7%)	
CSF NTTs				<0.001^a,b,c^
Reactive	149 (77.6%)	42 (55.3%)	2 (1.1%)	
Unreactive	43 (22.4%)	34 (44.7%)	181 (98.9%)	
CSF TTs				<0.001^b,c^
Reactive	183 (95.3%)	76 (100.0%)	48 (26.2%)	
Unreactive	9 (4.7%)	0 (0.0%)	135 (73.8%)	
Serum albumin (g/L)	38.10 (35.80, 40.38)	39.70 (36.30, 43.45)	38.60 (36.00, 41.30)	0.009^a^
Serum IgG (g/L)	11.25 (9.60, 13.98)	11.20 (9.38, 13.08)	11.20 (10.20, 13.30)	0.474
CSF glucose (mmol/L)	3.39 (2.99, 3.83)	3.40 (3.21, 3.88)	3.61 (3.22, 4.01)	0.004^b^
CSF chlorine (mmol/L)	126.00 (124.00, 128.00)	127.00 (125.00, 129.00)	125.00 (123.00, 127.00)	<0.001^b,c^
CSF protein (g/L)	0.79 (0.63, 1.06)	0.37 (0.32, 0.42)	0.39 (0.30, 0.49)	<0.001^a,b^
CSF nucleated cells (×10^-6^/L)	6.00 (1.00, 26.00)	0.00 (0.00, 1.75)	0.00 (0.00, 1.00)	<0.001^a,b^
CSF albumin (g/L)	0.35 (0.25, 0.49)	0.19 (0.15, 0.22)	0.21 (0.17, 0.30)	<0.001^a,b,c^
CSF IgG (g/L)	0.19 (0.10, 0.36)	0.06 (0.03, 0.10)	0.03 (0.02, 0.05)	<0.001^a,b,c^
Albumin quotient (×10^-3^)	9.21 (6.65, 12.55)	4.65 (3.73, 5.86)	5.57 (4.30, 7.70)	<0.001^a,b,c^
IgG quotient (×10^-3^)	16.71 (10.80, 30.19)	5.38 (3.22, 9.14)	3.01 (2.15, 4.37)	<0.001^a,b,c^
IgG index	1.82 (1.01, 2.80)	1.16 (0.68, 1.85)	0.52 (0.46, 0.61)	<0.001^a,b,c^
IgG synthesis rate	62.94 (30.73, 141.54)	13.21 (1.20, 32.28)	0.00 (0.00, 2.20)	<0.001^a,b,c^
Clinical diagnosis				<0.001^b,c^
Neurosyphilis	153 (79.7%)	53 (69.7%)	35 (19.1%)	
Non-neurosyphilis	39 (20.3%)	23 (30.3%)	148 (80.9%)	

There is a significant difference between each pair (*P* < 0.05):

a Class 1 vs. Class 2.

b Class 1 vs. Class 3.

c Class 2 vs. Class 3.

Albumin quotient is used to assess BBB permeability, reflecting conditions such as CNS infections; IgG quotient provides a rough indication of intrathecal IgG concentrations; the IgG index specifically determines the presence of intrathecal IgG synthesis in the CNS after excluding BBB effects; the IgG synthesis rate, based on a complex mathematical formula, precisely estimates the intrathecal IgG synthesis fraction.

TRUST, toluidine red unheated serum test; CSF, cerebrospinal fluid; TTs, treponemal tests; NTTs, non-treponemal tests; BBB, blood-brain barrier.

To investigate the association between LCA subtypes and traditional neurosyphilis classification, we conducted a correlation analysis on 35 neurosyphilis patients with confirmed traditional classification diagnoses. Among them, 28 typical neurosyphilis cases included 4 with syphilitic meningitis, 23 with general paresis, and 1 with tabes dorsalis; The 7 atypical neurosyphilis cases comprised 6 with general paresis and 1 with tabes dorsalis. Correlation analysis revealed no significant association between the two classifications (*P* > 0.05).

### 3.3 Construction of ML models

For machine learning model construction, six key predictive variables were selected: age, serum TRUST titer, CSF protein, CSF nucleated cells, IgG index, and CSF TTs. Multiple machine learning algorithms—including RF, XGBoost, GBDT, SVM, LR, and ANN—were systematically evaluated (**Table 3**). The XGBoost model demonstrated superior performance, achieving a high AUC (0.966), excellent discriminatory power, and substantial accuracy (0.873), with consistent performance between training and test sets. In contrast, other models exhibited variable test set performance: while RF attained the highest AUC (0.982), it showed lower accuracy (0.822); GBDT maintained comparable accuracy to XGBoost (0.870) but with a marginally lower AUC (0.947); whereas SVM, LR, and ANN displayed moderate performance without distinct advantages.

**Table 3:** Summary of performance metrics for the constructed ML models.

Models	Cohorts	AUC	Accuracy	Kappa	Sensitivity	Specificity	PPV	NPV
Random forest	Training	0.999	0.981	0.969	0.976	0.991	0.972	0.990
	Testing	0.966	0.855	0.768	0.845	0.925	0.840	0.930
XGBoost	Training	0.995	0.958	0.933	0.953	0.979	0.949	0.978
	Testing	0.966	0.873	0.797	0.862	0.934	0.855	0.935
GBDT	Training	0.995	0.965	0.945	0.967	0.984	0.951	0.982
	Testing	0.962	0.882	0.812	0.872	0.941	0.856	0.939
SVM	Training	0.993	0.934	0.894	0.900	0.965	0.931	0.970
	Testing	0.950	0.818	0.708	0.779	0.906	0.785	0.908
Logistic	Training	0.965	0.869	0.795	0.872	0.938	0.842	0.931
	Testing	0.929	0.827	0.730	0.838	0.915	0.804	0.909
ANN	Training	0.985	0.923	0.876	0.890	0.959	0.912	0.963
	Testing	0.937	0.827	0.727	0.818	0.913	0.799	0.909

AUC, area under the curve; PPV, positive predictive value; NPV, negative predictive value; XGBoost, eXtreme Gradient Boosting; GBDT, gradient boosting decision tree; SVM, support vector machines; ANN, artificial neural network.

### 3.4 Evaluation of the XGBoost model

**Figure 2:**
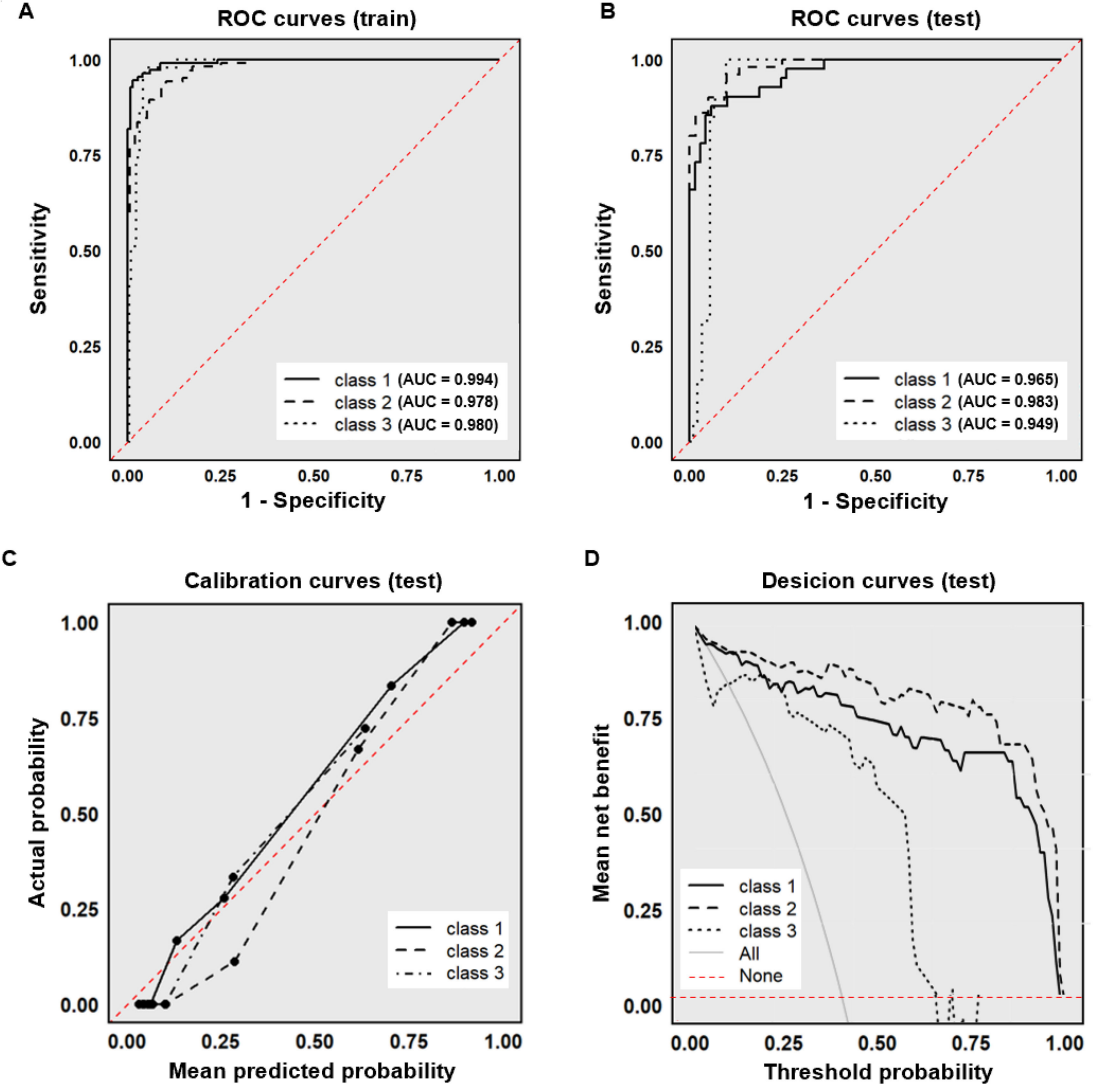
Performance of the XGBoost model. **(A)** ROC curve (training set); **(B)** ROC curve (test set); **(C)** Calibration curve (test set); **(D)** Decision curve (test set). The “All” curve represents the diagnostic benefit rate of blindly conducting examinations without classification. The “None” curve represents the diagnostic benefit rate of foregoing all examinations. ROC, receiver operating characteristic; AUC, area under the curve.

The constructed XGBoost model demonstrated robust discriminatory capacity across all three subtypes. In the training set, AUC values for Class 1 (typical neurosyphilis), Class 2 (atypical neurosyphilis), and Class 3 (non-neurosyphilis) were 0.994, 0.978, and 0.980, respectively (**Figure 2A**). In the internal test set, the model maintained high discriminative performance with AUCs of 0.965 (Class 1), 0.983 (Class 2), and 0.949 (Class 3) (**Figure 2B**). Calibration curves derived from the test set indicated excellent agreement between predicted probabilities and observed outcomes (**Figure 2C**). Decision curve analysis further confirmed substantial net benefit across different threshold probabilities, supporting the model’s utility for clinical decision-making (**Figure 2D**).

**Figure 3:**
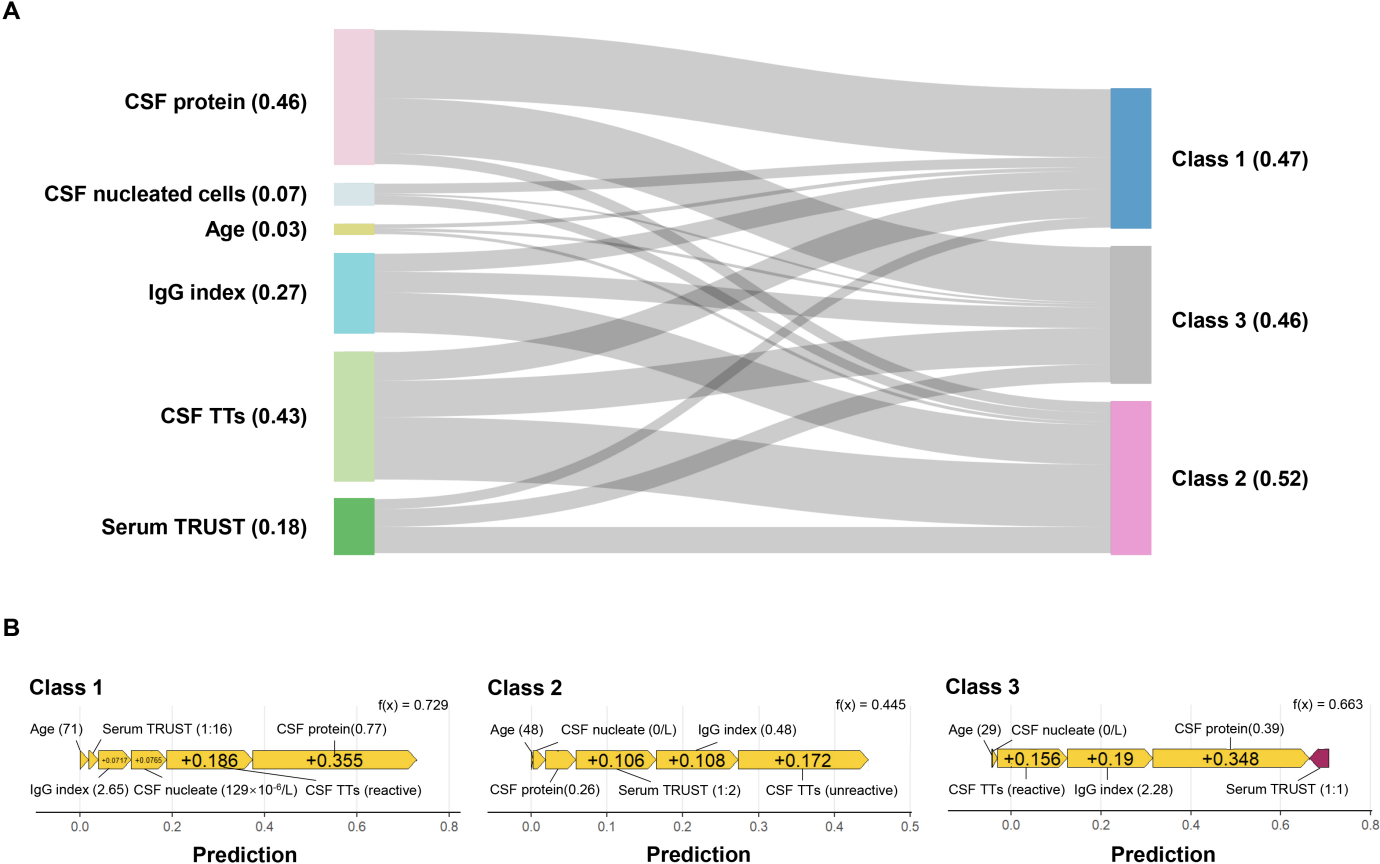
SHAP interpretation of the XGBoost model. **(A)** Sankey diagram of feature importance. The numerical values in parentheses represent SHAP values, and the thickness of the lines indicates the magnitude of each feature’s contribution to the target variable; **(B)** Force plots of representative sample features for class 1, class 2, and class 3. Yellow arrows denote support for the diagnosis of the corresponding class, while purple arrows indicate opposition to the diagnosis of the corresponding class, with the length of the arrows reflecting the magnitude of their contribution to the diagnosis. CSF, cerebrospinal fluid; TTs, treponemal tests; TRUST, toluidine red unheated serum test.

Further model interrogation was conducted using SHAP to elucidate the XGBoost decision framework. The feature importance analysis identified CSF protein as the predominant predictor, followed by CSF TTs, IgG index, serum TRUST titer, CSF nucleated cells, and age (**Figure 3A**). Subtype-specific contributions revealed that CSF protein substantially influenced the class 1 and class 2; CSF TTs, IgG index, and serum TRUST titer contributed significantly to class 2 and class 3. **Figure 3B** presents representative force plots visualizing individualized prediction mechanisms across all three subtypes.

**Figure 4:**
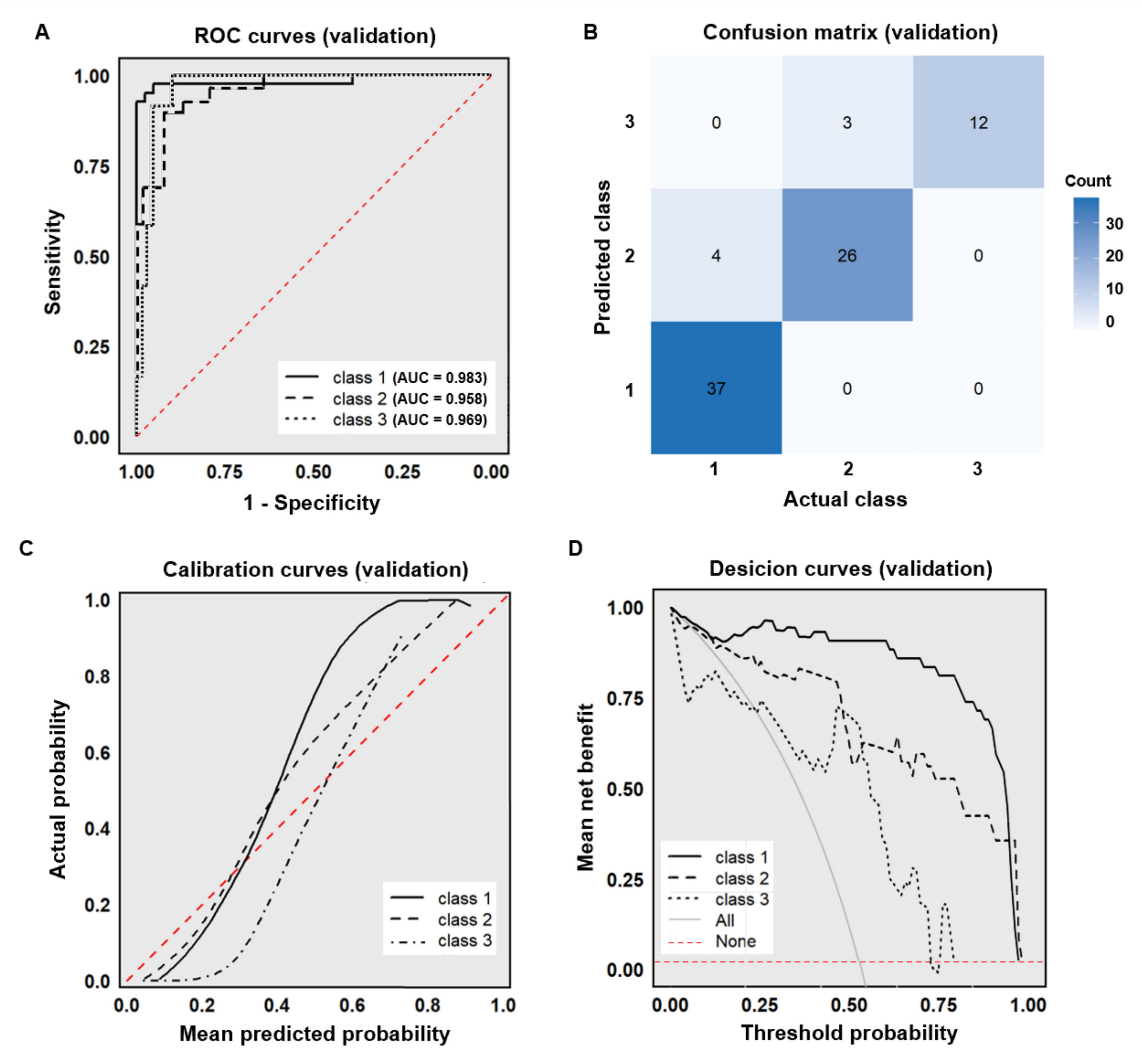
External dataset validation of the XGBoost model. **(A)** ROC curve (external validation set); **(B)** Confusion matrix (external validation set); **(C)** Calibration curve (external validation set); **(D)** Decision curve (external validation set).

Cohort 2, utilized for external validation, demonstrated comparable distributions to the model development cohort 1 across demographic characteristics, syphilis serological antibodies, clinical diagnosis, and LCA classifications, with no statistically significant differences observed (*P*>0.05; **Supplementary Table S3**). The XGBoost model achieved robust performance metrics on this independent validation set. The AUC, accuracy, sensitivity, specificity, PPV, and NPV were 0.970, 0.915, 0.933, 0.961, 0.889, 0.951, respectively (**Figure 4A**). Similarly, in the external validation set, the model demonstrated good predictive accuracy and clinical practicality (**Figures 4B-D**).

## 4 Discussion

The persistent global burden of syphilis infection underscores the growing importance of accurately identifying the neuroinvasive risk (Quilter et al., 2021). Current diagnostic approaches relying on CSF analysis and serological testing demonstrate limited sensitivity and specificity, particularly for atypical presentations, while treatment responses show marked heterogeneity across patients with different CSF characteristics (Shi M. et al., 2025). This study addresses this gap through the novel integration of LCA and ML algorithms.

LCA identified three clinically distinct subtypes among suspected neurosyphilis patients using seven key variables: typical neurosyphilis (class 1), atypical neurosyphilis (class 2), and non-neurosyphilis (class 3), which provides a classification basis for individualized treatment. Atypical neurosyphilis, comprising 17.9% of cases, is diagnostically challenging due to its nonspecific presentation of neuropsychiatric symptoms (e.g., mild cognitive decline, behavioral abnormalities, headaches), which often results in misdiagnosis as primary psychiatric or age-related conditions. These patients exhibit normal CSF protein (<0.5 g/L) and nucleated cell counts (<5×10^6^/L), with lower serum TRUST titers (<1:16) and distinct CSF immunological profiles (albumin quotient, IgG synthesis rate, IgG index) compared to typical neurosyphilis (*P* < 0.05) yet evidence of intrathecal antibody synthesis, suggests a distinct host-pathogen interaction. Importantly, persistent CSF protein elevation post-treatment in some patients suggests that atypical presentations may represent inherent disease variants rather than treatment artifacts (Dunaway et al., 2020).

Mechanistically, this atypical subtype may be associated with the immune privilege of the central nervous system, where pathogens entering the parenchyma may escape systemic immunological recognition and fail to effectively trigger a strong local inflammatory response (Enzmann et al., 2018). Another interpretation is that this may represent a chronic or late-stage infection, where the initial inflammation has subsided, but the persistent presence of antigens continues to drive the production of antibody and the antibiotics concentration in CSF should be tracked. Unlike typical cases, who exhibit a compromised BBB and influx of various peripheral immune cells triggering a more intense inflammatory response, atypical neurosyphilis maintains a relatively intact BBB (Prinz and Priller, 2017), with intrathecal immunoglobulin production driven by B cells recruited through chemokines (Yu et al., 2017). In addition, studies have shown that the levels of CSF protein and white blood cell count are positively correlated with the inflammatory markers CXCL13, IL-6, and IL-10, which suggests that patients with atypical neurosyphilis may not have a significant or active inflammatory response (Dersch et al., 2015; Yan et al., 2017).

The analysis indicated that LCA-derived subtypes were not correlated with traditional classifications. Traditional classifications require integration of clinical manifestations, imaging examinations, and laboratory tests, and display the affected sites, whereas LCA-derived subtypes are based on simple laboratory indicators required for routine diagnosis, which are more accessible and affordable, and can be explained by biological-level mechanisms. Actually, no evidence supports subtype-specific treatments (Ropper, 2019; Chen et al., 2025). However, patients with the typical subtype exhibit more pronounced inflammation, potentially necessitating future validation of combining antibiotics with anti-inflammatory therapies (such as corticosteroids). Emerging evidence suggests variations in neuroinvasiveness among *Treponema pallidum* genotypes both in rabbit models and humans (Tantalo et al., 2005; Marra et al., 2010). Future investigations should explore correlations between LCA subtypes and strain genotypes to elucidate the clinical significance of LCA subtypes and molecular mechanisms underlying neuroinvasion.

For ML model construction, six easily obtainable and objective laboratory variables (age, serum TRUST titer, CSF protein, CSF nucleated cells, IgG index, CSF TTs) were selected to .capture nonlinear biological relationships (Stahlschmidt et al., 2022). Among them, serum TRUST titer, CSF TTs, and CSF protein simultaneously serve as variables for determining LCA classification; additionally, CSF protein and nucleated cells - also identified as key predictors in Zou et al.’s diagnostic model (the minimum value of AUC: 0.84) - demonstrated particular importance in our XGBoost algorithm (Zou et al., 2023). Studies indicate that TPPA and FTA-ABS exhibit comparable diagnostic sensitivity for neurosyphilis, allowing institutions to select appropriate CSF TTs based on their specific circumstances (Marra et al., 2017; Park et al., 2020). Older age was the independent risk factor for HIV-negative neurosyphilis patients, though it remains uncertain whether this association stems from age-related immune system changes or disease courses (Shi et al., 2016). Furthermore, intrathecal B-cell enrichment and immunoglobulin production have been observed in neurosyphilis patients, establishing the IgG index as both a novel diagnostic and disease progression indicator (Yu et al., 2017). The IgG index is a computational indicator with low cost, requiring only respective measurements of albumin and IgG levels in serum and CSF.

As an optimized algorithm based on GBDT, XGBoost serially trains multiple weak learners, where each tree attempts to correct the prediction errors of the preceding one, thereby progressively optimizing the model, and has exhibited excellent predictive performance in medical applications (Shi J. et al., 2025). Through evaluation using ROC analysis, calibration curves, and decision curves, it was found that the XGBoost model demonstrated favorable discriminative and calibration capabilities in predicting neurosyphilis (AUC of 0.966 on the internal test set and 0.970 on the external validation set). According to the validation set data, the XGBoost model showed relatively low prediction accuracy and clinical decision-making benefit for non-neurosyphilis cases, and it was prone to misclassifying atypical neurosyphilis patients as non-neurosyphilis patients (attributable to overlapping CSF protein/nucleated cell count profiles with non-neurosyphilis). However, all 3 misclassified cases had positive CSF TTs despite negative CSF NTTs, underscoring the critical need to confirm with CSF TTs when CSF NTTs are negative, given the latter’s known lower sensitivity (Satyaputra et al., 2021). In clinical practice, the usefulness of predictive models depends not only on their accuracy but also on their interpretability (Lancashire et al., 2025). SHAP analysis revealed CSF protein and CSF TTs as top predictors consistent with findings from relevant studies. Proteomics research supports the presence of characteristic inflammatory protein markers in the CSF or brain tissue of patients with neurosyphilis (Li et al., 2024; Zhang et al., 2025). Meanwhile, elevated CSF protein levels may indicate disease progression, the severity of neurosyphilis, and poor prognosis (Chen et al., 2025). While CSF TTs exhibit limited specificity due to blood-CSF barrier permeability, high titers (CSF TPPA≥1:320 or ≥1:640) remain diagnostically valuable (Marra et al., 2017; Park et al., 2020; Shi M. et al., 2025).

This study has certain limitations. Firstly, as follow-up information and other relevant data were not included in this study, it is challenging to establish a clear association between the LCA subtypes and their long-term clinical prognostic significance. In the future, it is necessary to track the treatment status, treatment efficacy, and recurrence rate of patients with different subtypes by prospective studies with long-term follow-up. Secondly, these models were trained and evaluated based on data from a single center. It should be noted that our center is a large tertiary hospital that mainly treats complex and comorbid cases, which may introduce population bias. Therefore, population differences should be carefully considered in future research, and multicenter validation is needed to enhance generalizability. Thirdly, the external validation cohort (cohort 2) consisted of patients from the same center but at different time points, which may further limit the generalizability of the findings.

In summary, this study establishes a clinically actionable framework for neurosyphilis diagnosis through the innovative LCA of routine clinical indicators. Our approach successfully differentiates neurosyphilis from non-neurosyphilis cases while further classifying neurosyphilis into two clinically distinct subtypes - typical and atypical forms. The developed XGBoost-based clinical decision support system demonstrates robust performance in subtype identification, enabling precise management of this diagnostically challenging condition.

## Data Availability

The original contributions presented in the study are included in the article/**Supplementary Material**. Further inquiries can be directed to the corresponding author.
