# Supplementary material for: Latent class analysis and machine learning for clinical subtyping prediction and differentiation in suspected neurosyphilis patients
Source: Front Cell Infect Microbiol. 2025 Nov 25;15:1665468. doi: 10.3389/fcimb.2025.1665468 (PMC12685824; doi:10.3389/fcimb.2025.1665468)
Supplement: Supplementary file 1 [file DataSheet1.pdf]

frontiers

Supplementary Material

1 Supplementary Methods

1.1 Variable transformation criteria for latent class analysis (LCA)

All variables were dichotomized with "1" representing positive findings and "2" indicating negative results. Based on established literature, age ≥45 years (assigned as "1") and male sex (assigned as "1") were identified as risk factors for neurosyphilis in HIV-negative patients with primary, secondary, or latent syphilis [1]. Serum TRUST titer ≥1:16 (assigned as "1") was similarly classified as a neurosyphilis risk factor in HIV-negative populations [2]. Diagnostic thresholds (Guidelines for diagnosis and treatment of syphilis, gonorrhea and genital *Chlamydia trachomatis* infection, 2020) were applied to define abnormal CSF parameters: CSF protein ≥0.5 g/L (assigned as "1") and CSF nucleated cells $\geq 5\times10^6$/L (assigned as "1"). Laboratory-specific reference ranges determined positivity (assigned as "1") for serum IgG (≥15.5 g/L), serum albumin (≥35 g/L), CSF glucose (≥4.4 mmol/L), CSF chloride (≥130 mmol/L), CSF IgG (≥0.041 g/L), CSF albumin (≥0.337 g/L), IgG index (≥0.84), and IgG synthesis rate (≥5.81). Thresholds for intrathecal synthesis markers were derived from receiver operating characteristic (ROC) analysis using Youden's index: IgG quotient ≥0.006109 (assigned as "1") and albumin quotient ≥0.007138 (assigned as "1"). CSF treponemal tests (TTs) were considered positive (assigned as "1") if either TPPA or FTA-ABS was reactive, while CSF non-treponemal tests (NTTs) were positive (assigned as "1") for TRUST or VDRL reactivity.

1.2 Optimal variable selection for LCA

Seventeen candidate variables were evaluated: age, sex, serum TRUST titer, serum IgG, serum albumin, CSF TTs, CSF NTTs, CSF protein, CSF nucleated cells, CSF glucose, CSF chloride, CSF IgG, CSF albumin, IgG quotient, albumin quotient, IgG index, and IgG synthesis rate. Variable combinations were systematically assessed based on the coverage and redundancy of four clinical domains: (1) risk factors for neurosyphilis, (2) diagnostic criteria, (3) intrathecal humoral immune activation indicators, and (4) blood-brain barrier injury indicators, as well as the correlation between each variable and neurosyphilis. Fifteen variable combinations were screened out. The akaike information criterion (AIC), bayesian information criterion (BIC), and entropy values for each combination were generated in batch (Table S1). Subsequently, the optimal variable combination was selected by considering the balance of class proportions and model interpretability.

2 Supplementary Figures and Tables

Supplementary Table S1. Model fit statistics for selected variable combinations.

Variables	Class	AIC	BIC	Likelihood	Entropy
Serum TRUST, age, CSF nucleate, IgG synthesis rate, Qalb, CSF TTs, CSF NTTs	2	3664.809	3726.481	-1817.405	0.827
	3	3634.138	3728.702	-1794.069	0.812

	4	3597.499	3724.954	-1767.749	0.835
	5	3594.022	3754.369	-1758.011	0.831
Serum TRUST, age, CSF protein, CSF nucleate, IgG synthesis rate, Qalb, CSF TTs, CSF NTTs	2	4158.527	4228.422	-2062.264	0.818
	3	4063.772	4170.670	-2005.886	0.816
	4	3993.395	4137.296	-1961.697	0.828
	5	3982.268	4163.172	-1947.134	0.799
Serum TRUST, age, CSF protein, IgG synthesis rate, Qalb, CSF TTs, CSF NTTs	2	3681.429	3743.101	-1825.714	0.819
	3	3602.839	3697.403	-1778.420	0.836
	4	3530.483	3657.939	-1734.242	0.838
	5	3530.946	3691.294	-1726.473	0.850
Serum TRUST, CSF nucleate, IgG synthesis rate, Qalb, CSF TTs, CSF NTTs	2	3090.255	3143.704	-1532.128	0.819
	3	3045.998	3128.228	-1502.999	0.981
	4	3017.148	3128.158	-1481.574	0.836
	5	3025.840	3165.630	-1478.920	0.850
Serum TRUST, CSF protein, CSF nucleate, IgG synthesis rate, Qalb, CSF TTs, CSF NTTs	2	3582.900	3644.572	-1776.450	0.818
	3	3487.554	3582.118	-1720.777	0.809
	4	3420.669	3548.125	-1679.335	0.820
	5	3411.848	3572.195	-1666.924	0.788
Serum TRUST, CSF protein, IgG synthesis rate, Qalb, CSF TTs	2	2669.314	2714.541	-1323.657	0.788
	3	2626.264	2696.159	-1296.132	0.793

	4	2582.614	2677.178	-1268.307	0.736
	5	2589.563	2708.796	-1265.782	0.796
Serum TRUST, CSF protein, IgG synthesis rate, Qalb, CSF TTs, CSF NTTs	2	3106.432	3159.881	-1540.216	0.816
	3	3017.824	3100.053	-1488.912	0.875
	4	2957.570	3068.579	-1451.785	0.837
	5	2964.830	3104.619	-1448.415	0.765
Serum TRUST, CSF protein, IgG synthesis rate, Qalb, CSF TTs, CSF TTs	2	2660.031	2705.257	-1319.016	0.773
	3	2596.624	2666.519	-1281.312	0.793
	4	2569.175	2663.739	-1261.588	0.758
	5	2579.126	2698.358	-1260.563	0.752
Serum TRUST, gender, age, CSF nucleate, IgG synthesis rate, CSF protein, CSF TTs, CSF NTTs	2	4138.552	4208.447	-2052.276	0.821
	3	4087.066	4193.965	-2017.533	0.759
	4	4036.270	4180.171	-1983.135	0.792
	5	4028.843	4209.747	-1970.421	0.804
Serum TRUST, gender, age, CSF nucleate, IgG synthesis rate, Qalb, CSF TTs, CSF NTTs	2	4199.199	4269.094	-2082.599	0.817
	3	4164.167	4271.065	-2056.083	0.826
	4	4131.714	4275.616	-2030.857	0.754
	5	4119.247	4300.152	-2015.624	0.779
Serum TRUST, gender, age, CSF protein, CSF nucleate, IgG synthesis rate, Qalb, CSF TTs, CSF NTTs	2	4691.347	4769.465	-2326.674	0.821
	3	4592.612	4711.844	-2267.306	0.826
	4	4518.004	4678.351	-2220.002	0.815

	5	4510.695	4712.157	-2206.348	0.792
Serum TRUST, gender, age, CSF protein, IgG synthesis rate, Qalb, CSF TTs, CSF NTTs	2	4208.963	4278.858	-2087.481	0.817
	3	4122.789	4229.688	-2035.395	0.848
	4	4049.990	4193.891	-1989.995	0.831
	5	4046.677	4227.582	-1979.338	0.847
Serum TRUST, gender, CSF nucleate, IgG synthesis rate, Qalb, CSF TTs, CSF NTTs	2	3623.377	3685.049	-1796.688	0.812
	3	3584.506	3679.070	-1769.253	0.823
	4	3550.135	3677.590	-1744.067	0.766
	5	3546.134	3706.481	-1734.067	0.747
Serum TRUST, gender, CSF protein, CSF nucleate, IgG synthesis rate, Qalb, CSF TTs, CSF NTTs	2	4114.736	4184.631	-2040.368	0.822
	3	4016.428	4123.326	-1982.214	0.823
	4	3945.467	4089.368	-1937.733	0.810
	5	3940.666	4121.570	-1926.333	0.769
Serum TRUST, gender, CSF protein, IgG synthesis rate, Qalb, CSF TTs, CSF NTTs	2	3632.655	3694.327	-1801.328	0.815
	3	3547.981	3642.544	-1750.990	0.849
	4	3477.519	3604.975	-1707.760	0.829
	5	3483.473	3643.820	-1702.736	0.841

TRUST, toluidine red unheated serum test; Qalb, albumin quotient; CSF, cerebrospinal fluid; TTs, treponemal tests; NTTs, non-treponemal tests.

Supplementary Table S2. Variable selection criteria for machine learning (ML).

	Gender	Age	Serum TRUST	CSF protein	CSF nucleate	CSF glucose	CSF cholrine	Serum IgG	Serum albumin
LASSO	Y	Y	Y	Y	Y	N	Y	Y	Y
Boruta	Y	Y	Y	Y	Y	N	Y	N	Y
Statistical significance	N	Y	Y	Y	Y	Y	Y	N	Y
Literature to support	Y	Y	Y	Y	Y	N	N	N	N

	CSF IgG	CSF albumin	IgG index	IgG synthesis rate	IgG quotient	Albumin quotient	CSF NTTs	CSF TTs
LASSO	Y	Y	Y	Y	N	N	Y	Y
Boruta	Y	Y	Y	Y	Y	N	Y	Y
Statistical significance	Y	Y	Y	Y	Y	Y	Y	Y
Literature to support	N	N	Y	Y	N	N	Y	Y

TRUST, toluidine red unheated serum test; CSF, cerebrospinal fluid; TTs, treponemal tests; NTTs, non-treponemal tests; LASSO, least absolute shrinkage and selection operator.

Supplementary Table S3. Demographic, clinical, and laboratory features between cohort 1 and cohort 2.

	Cohort 1	Cohort 2	*P*
N	369	82	
Age (y)	49.75±14.95	49.34±12.72	0.818
Gender			0.205
Male	248 (67.2%)	61 (74.4%)	

Female	121 (32.8%)	21 (25.6%)	
Serum TRUST titer			0.165
≥1:16	146 (39.6%)	39 (47.6%)	
<1:16	223 (60.4%)	43 (52.4%)	
CSF NTTs			0.266
Reactive	153 (41.5%)	40 (48.8%)	
Unreactive	216 (58.5%)	42 (51.2%)	
CSF TTs			0.273
Reactive	247 (66.9%)	60 (73.2%)	
Unreactive	122 (33.1%)	22 (26.8%)	
Clinical diagnosis			0.205
Neurosyphilis	192 (52.0%)	49 (59.8%)	
Non-neurosyphilis	177 (48.0%)	33 (40.2%)	
Predicted classes			0.323
Class 1	151 (40.9%)	41 (50.0%)	
Class 2	154 (41.7%)	29 (35.4%)	
Class 3	64 (17.3%)	12 (14.6%)	

TRUST, toluidine red unheated serum test; CSF, cerebrospinal fluid; TTs, treponemal tests; NTTs, non-treponemal tests.

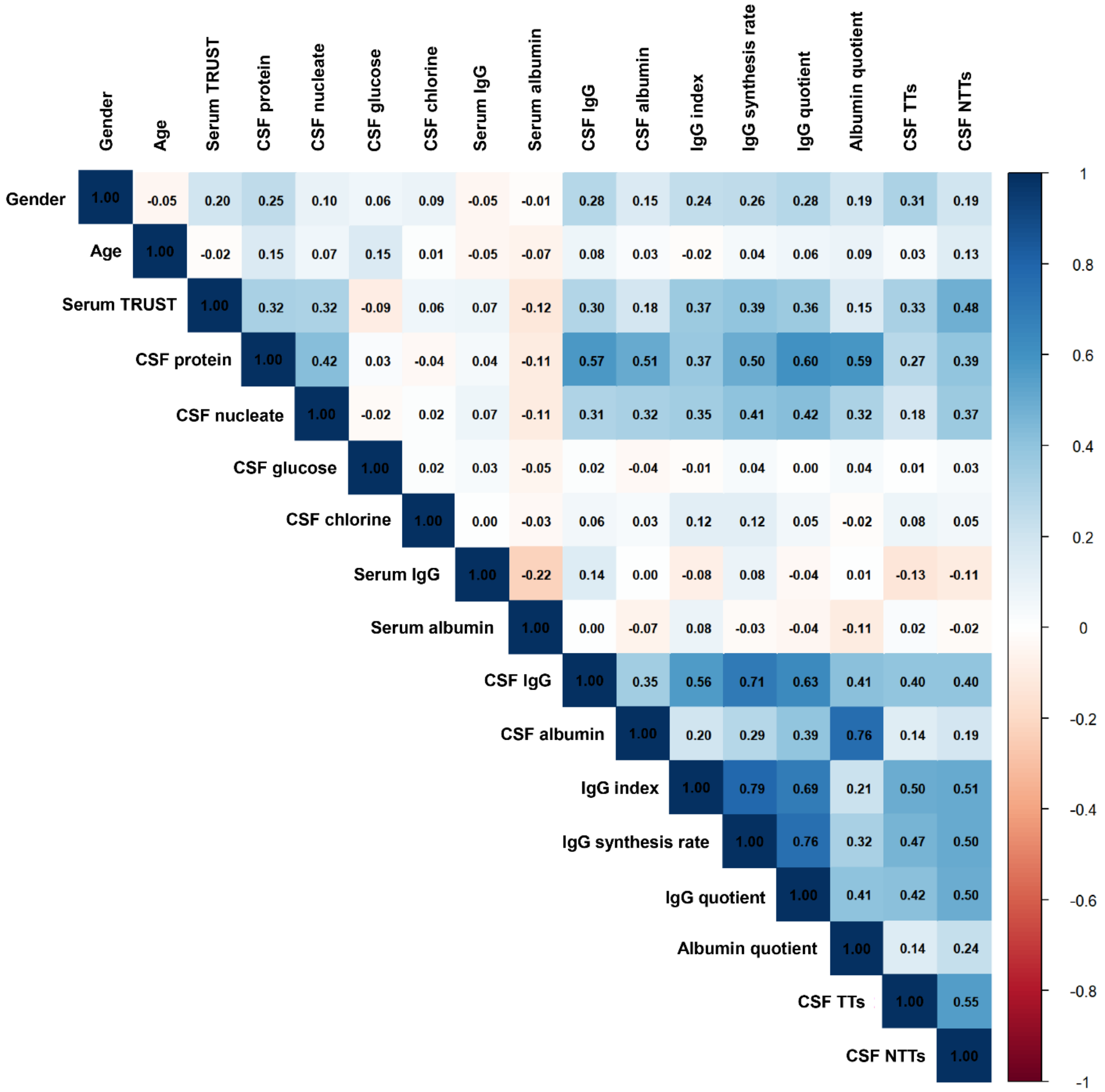

Supplementary Figure S1. Correlation matrix heatmap for LCA variable selection.

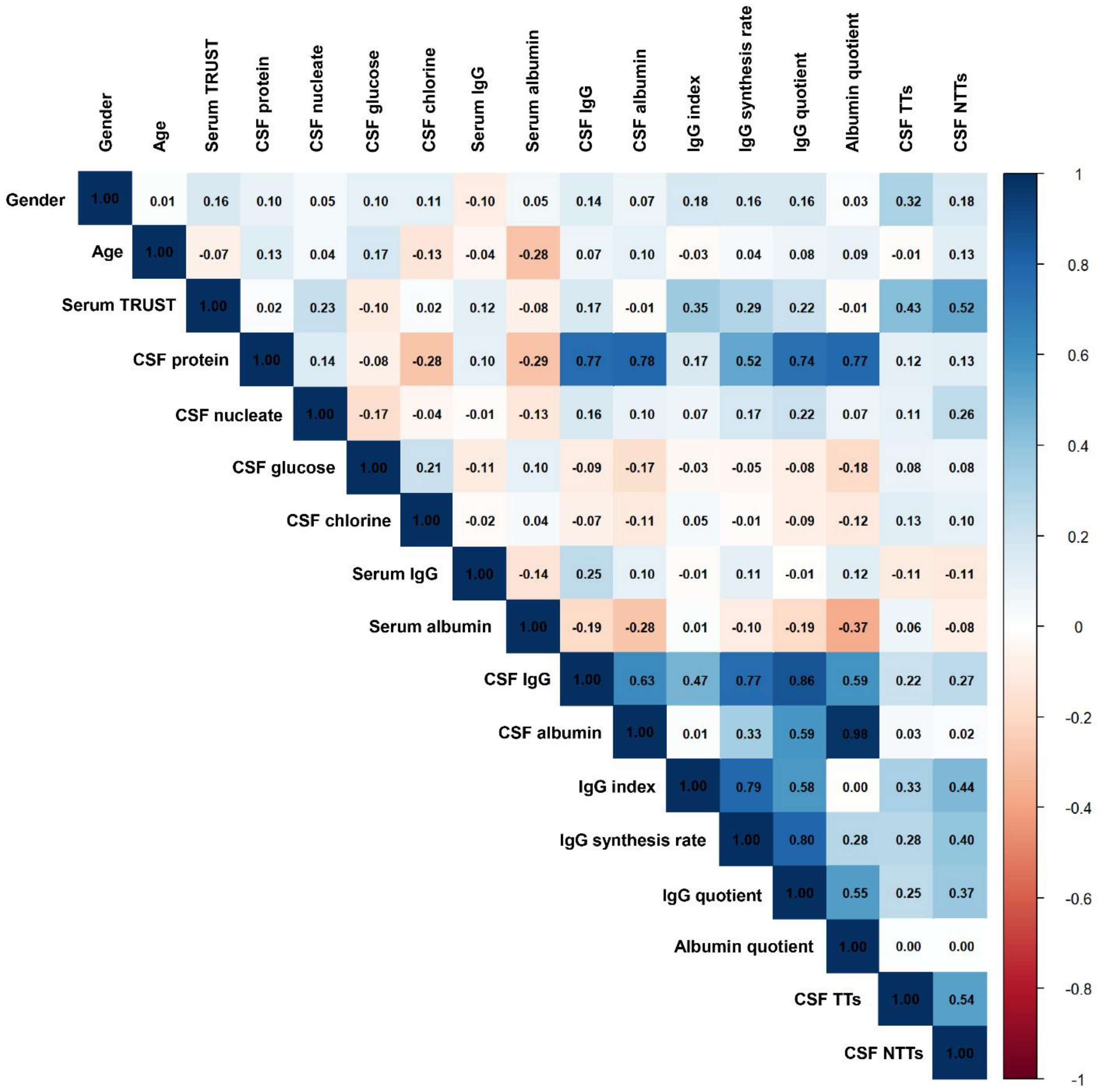

Supplementary Figure S2. Correlation matrix heatmap for ML variable selection.

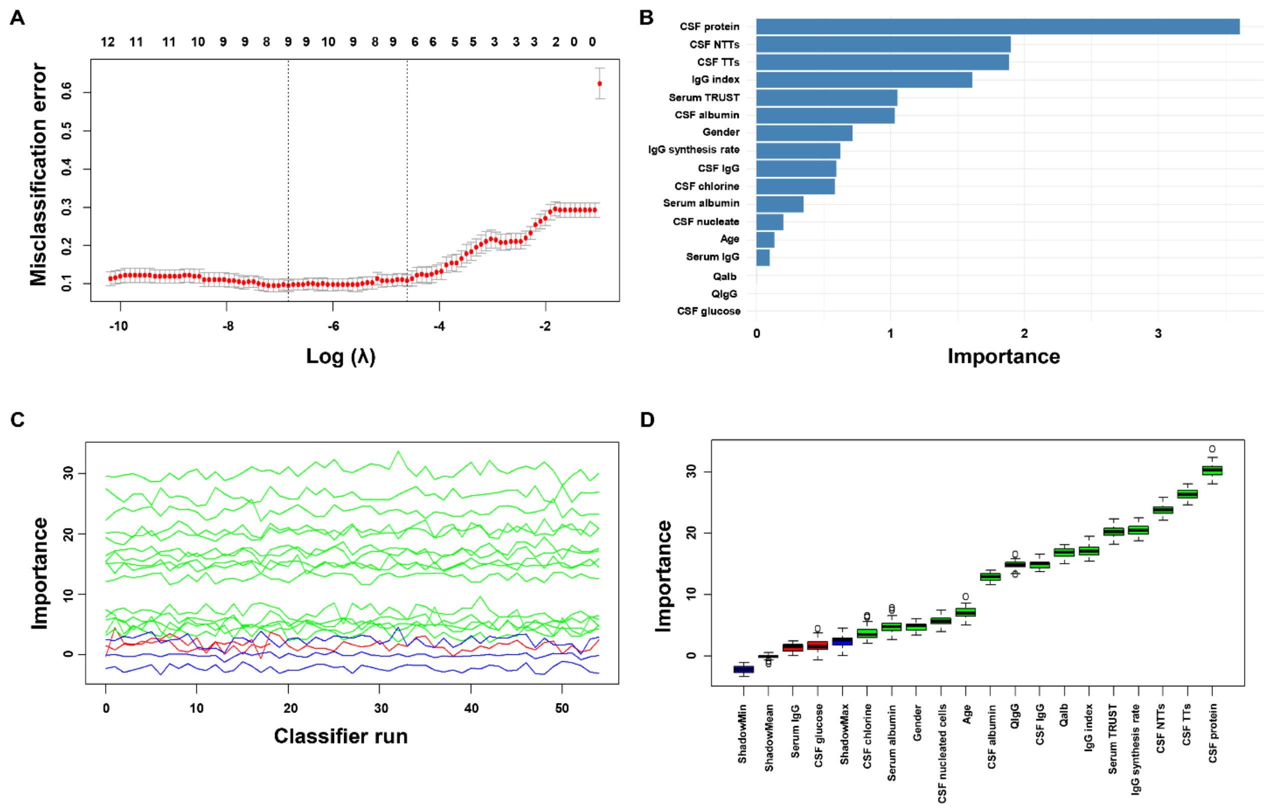

Supplementary Figure S3. LASSO regression and Boruta algorithm for screening variables in ML. **(A)** Selection of λ in the LASSO regression using 10-fold cross-validation. The doted vertical lines are set usine the minimum criteria and the one standard erroof the minimum criteria; **(B)** The variable importance of LASSO regression; **(C)** Changes in the importance scores of each variable in Boruta algorithm; **(D)** The variable importance of Boruta algorithm.
